# Inbreeding depression under mixed outcrossing, self-fertilization and sib-mating

**DOI:** 10.1186/s12862-016-0668-2

**Published:** 2016-05-17

**Authors:** Emmanuelle Porcher, Russell Lande

**Affiliations:** Centre d’Ecologie et des Sciences de la Conservation (UMR7204), Sorbonne Universités, MNHN, CNRS, UPMC, 57 rue Cuvier, Paris, 75005 France; Department of Life Sciences, Imperial College London, Ascot, Berkshire UK

**Keywords:** Mixed mating, Self-fertilization, Biparental inbreeding, Nearly recessive lethals, Mutation, Inbreeding depression

## Abstract

**Background:**

Biparental inbreeding, mating between two relatives, occurs at a low frequency in many natural plant populations, which also often have substantial rates of self-fertilization. Although biparental inbreeding is likely to influence the dynamics of inbreeding depression and the evolution of selfing rates, it has received limited theoretical attention in comparison to selfing. The only previous model suggested that biparental inbreeding can favour the maintenance of stable intermediate selfing rates, but made unrealistic assumptions about the genetic basis of inbreeding depression. Here we extend a genetic model of inbreeding depression, describing nearly recessive lethal mutations at a very large number of loci, to incorporate sib-mating. We also include a constant component of inbreeding depression modelling the effects of mildly deleterious, nearly additive alleles. We analyze how observed rates of sib-mating influence the mean number of heterozygous lethals alleles and inbreeding depression in a population reproducing by a mixture of self-fertilization, sib-mating and outcrossing. We finally use the ensuing relationship between equilibrium inbreeding depression and population selfing rate to infer the evolutionarily stable selfing rates expected under such a mixed mating system.

**Results:**

We show that for a given rate of inbreeding, sib-mating is more efficient at purging inbreeding depression than selfing, because homozygosity of lethals increases more gradually through sib-mating than through selfing. Because sib-mating promotes the purging of inbreeding depression and the evolution of selfing, our genetic model of inbreeding depression also predicts that sib-mating is unlikely to maintain stable intermediate selfing rates.

**Conclusions:**

Our results imply that even low rates of sib-mating affect plant mating system evolution, by facilitating the evolution of selfing via more efficient purging of inbreeding depression. Alternative mechanisms, such as pollination ecology, are necessary to explain stable mixed selfing and outcrossing.

## Background

Inbreeding plays a central role in the evolution of many plant and animal populations, affecting for example effective population size [32], the speed of adaptation [15], or the accumulation of deleterious mutations and the resulting inbreeding depression [27, 33]. Much attention so far has focused on self-fertilization, the most extreme form of inbreeding, which is widespread in plant populations [16], and also occurs in some hermaphroditic animals [21]. However, biparental inbreeding (BI), or mating between two relatives, is also likely to occur frequently in natural populations due to small local population size [46, 48], limited dispersal causing fine-scale genetic structure [26, 47, 49] or social structure in animals [41]. After Ritland [38] developed a relatively simple method to estimate BI using genotypes for molecular markers in progeny arrays, by comparing multi-locus vs. single-locus estimates of outcrossing rates, large datasets on BI have accumulated in plant populations. Since the first analysis of such data [9] BI has been demonstrated to occur frequently, albeit at low rates.

In self-compatible hermaphroditic plant populations, BI acts along with selfing to diminish the mean number of recessive deleterious mutations and the inbreeding depression maintained by a balance between mutation and selection, as it increases the inbreeding coefficient of the population, exposing recessive deleterious mutations to selection in homozygous form. Several experimental observations confirm that BI can contribute to purging inbreeding depression [6, 19, 22, 39, 44]. The only previous theoretical treatment of the joint influence of selfing and BI on deleterious mutations [42] showed that such purging by BI could facilitate the evolution of increased selfing rates. Uyenoyama [42] also suggested that BI could promote the maintenance of stable mixed mating systems with intermediate selfing rates when inbreeding depression is low (<0.5), which would provide a general answer to the “enigma" of mixed mating systems [16]. However, this prediction may strongly depend on a number of simplifying assumptions regarding inbreeding depression, its genetic basis and its expression in individuals produced by BI. First, when inbreeding depression was allowed to evolve with the mating system in [42], it was modelled assuming completely recessive mildly deleterious mutations. However, the distribution of fitness effects of mutations is known to be strongly bimodal (e.g. [10]), such that inbreeding depression is often caused by a combination of nearly recessive highly deleterious (lethal and semi-lethal) mutations and moderately recessive or nearly additive mildly deleterious mutations (reviewed in [5]). These two classes of mutations exhibit contrasting responses to natural selection in an inbred population [3]. Second, stable mixed mating systems in Uyenoyama’s model were only studied assuming zero inbreeding depression in offspring produced by BI and constant inbreeding depression associated with selfing, neither of which is biologically plausible based on the available empirical data (see above and [45]).

Here, we develop a more realistic genetic model to analyze how sib-mating, a relatively common form of BI, influences inbreeding depression in a population reproducing by a mixture of self-fertilization and outcrossing, a necessary first step to make predictions on the evolution of selfing rates. Our model differs from [42] in the genetic basis of inbreeding depression and includes the two aforementioned components. The component of inbreeding depression due to moderately recessive mildly deleterious mutations is unlikely to be purged much by inbreeding because exposure to selection depends little on inbreeding for nearly additive mutations. It was modelled as a constant, background component of inbreeding depression (as in [33]). The component of inbreeding depression due to highly deleterious mutations was modelled using the approach of Kondrashov [25] to describe evolution of deleterious mutations at a very large number of unlinked loci. We employed a simplified version of the Kondrashov model for nearly recessive lethal mutations [28]. The Kondrashov model accounts for zygotic disequilibrium (non-random associations of diploid genotypes among loci) caused by a mixture of selfing and outcrossing. These models assume infinite population size and individually rare deleterious mutations, which can become homozygous only through selfing; in their original form, they therefore cannot deal with sib-mating. We extend the Kondrashov model for nearly recessive lethals [28] by tracking three consecutive generations (grandparents, parents and offspring) to incorporate sib-mating.

This model allows us to study the effect of realistic levels of sib-mating on the equilibrium inbreeding depression in plant populations reproducing by a mixture of selfing and outcrossing. The resulting relationship between inbreeding depression and the population selfing rate is then used to infer the evolutionarily stable selfing rates expected in a population practising sib-mating, when inbreeding depression is allowed to evolve with the mating system. This is the second major difference from the approach of Uyenoyama [42], who assumed constant inbreeding depression upon selfing, independent of the selfing rate in the population. We show below that for a given rate of inbreeding sib-mating is more efficient than selfing in purging inbreeding depression; sib-mating is therefore expected to promote the evolution of selfing and is unlikely to maintain stable intermediate selfing rates.

## Results and discussion

**Fig. 1:**
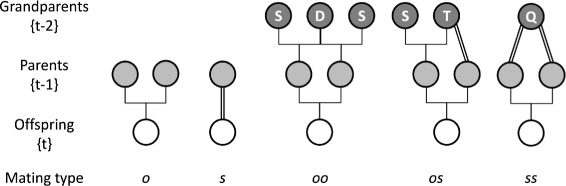
Pedigrees of types of crosses. *o*: outcrossing between unrelated individuals; *s*: selfing; *oo*: sib-mating between two outcrossed individuals; *os*: sib-mating between one outcrossed and one selfed individuals; *ss*: sib-mating between two selfed individuals. In the grandparental generation, letters S, D, T and Q refer to the single, double, triple and quadruple grandparents

In a population reproducing by a mixture of self-fertilization, outcrossing between unrelated individuals and sib-mating, the model calculates the mean number of nearly recessive heteroygous lethals, with dominance coefficient *h*=0.02, maintained per diploid genome at mutation-selection equilibrium under a genomic mutation to lethals *U*. In the following, $\bar {s}$ is the population primary selfing rate, at fertilization. The total inbreeding depression *δ* is defined as the decrease in mean fitness of selfed offspring vs. those produced by outcrossing between unrelated parents. The distribution of heterozygous lethals, combined with a constant (non-evolving) background inbreeding depression *d*=0.25 for selfing, produces the total inbreeding depression upon selfing. Individuals produced by different types of mating are denoted using subscripts without brackets: *o*, *s*, *oo*, *os*, and *ss*, respectively indicating outcrossing to an unrelated individual, self-fertilization, and sib-mating involving two outcrossed parents, one outcrossed and one selfed parent, or two selfed parents (Fig. 1). For more details, see Methods.

### Rates of BI in natural plant populations

**Fig. 2:**
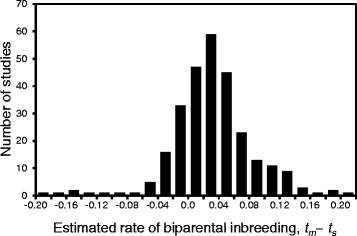
Distribution of the estimated rates of biparental inbreeding (BI) in natural plant populations

We first tested our assumption of a low frequency of BI (below 0.1), which was required to neglect the probability of BI occurring in two successive generations in any given lineage in the population. We merged two existing databases compiling multilocus estimates of outcrossing rates (*t*_*m*_) in natural plant populations [9, 16], retaining only species for which both multi-locus and mean single-locus (*t*_*s*_) estimates were available (276 species), and we estimated the frequency of BI from *t*_*m*_−*t*_*s*_ [38]. As expected, BI was generally rare in natural plant populations (Fig. 2), with an average value of 0.033 (90 *%* range [−0.038,0.144]). Less than 10 *%* of species have estimated rates of BI above 0.1, and many of these are likely due to sampling error, as obviously are all the negative estimates; the highest estimate of 0.218 [30] is from an agricultural population (seed orchard).

### Influence of sib-mating on mean number of lethals and total inbreeding depression

**Fig. 3:**
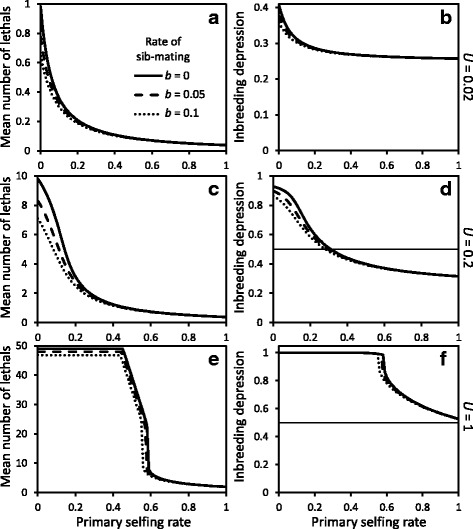
Mean number of heterozygous lethals at equilibrium (**a**, **c** & **e**) and average inbreeding depression (**b**, **d** & **f**) as a function of population selfing rate, for different rates of sib-mating *b* and genomic mutation rates to lethals *U*. Background inbreeding depression is *d*=0.25. On panels **d** and **f**, the thin horizontal line at 0.5 represents the automatic advantage of selfing, i.e. the threshold value for inbreeding below which evolution of increased selfing is favored

In a mixed-mating population without sib-mating, the mean number of deleterious mutations at equilibrium, and hence inbreeding depression, decreases with increased selfing rate (Fig. 3) due to increased homozygosity exposing recessive deleterious mutations to selection [27]. The decrease in mean lethals and inbreeding depression is relatively smooth under small or moderate genomic mutation rates to lethals (*U*=0.02 and *U*=0.2, Fig. 3a-d). At a high genomic mutation rate to lethals (*U*=1, Fig. 3e-f) there appears a fairly sharp purging threshold due to zygotic disequilibria and selective interference among lethals causing the secondary selfing rate to remain close to zero for primary selfing rates below the purging threshold [28]. The shape of the relationship between population selfing rate and the mean number of heterozygous lethals or total inbreeding depression at equilibrium remains qualitatively the same in the absence of background inbreeding depression (cf. Figure 5 in Appendix). Background inbreeding depression increases total inbreeding depression at equilibrium, and shifts the threshold selfing rates for purging recessive lethals towards higher values.

With sib-mating, the general pattern of decreased equilibrium number of lethals and inbreeding depression with increased selfing rate remains the same, but sib-mating can have a significant effect on the inbreeding depression and the purging threshold (Fig. 3). In a completely outcrossing population, sib-mating decreases the mean number of lethals to the greatest extent for small to moderate genomic mutation rates to lethals, *U*; in such populations with *U*=0.02, 0.2 or 1 the proportional decrease in the equilibrium number of heterozygous lethals caused by 10 % sib-mating is 0.34, 0.27 or 0.05 respectively. This pattern remains qualitatively the same without background inbreeding depression (Figure 5 in Appendix).

For a given rate of inbreeding, sib-mating is more efficient than selfing at purging inbreeding depression. This can be illustrated by comparing populations with identical expected inbreeding coefficient prior to selection, for example an outcrossing population with *b*=10 *%* half-sib (*oo*) mating vs. a predominantly outcrossing population with a selfing rate of 0.025 (without sib-mating); the equilibrium number of heterozygous lethals is always lower in the population with sib-mating than in the partly selfing population, and the difference increases at higher genomic mutation rates to lethals (0.65 vs. 0.70, 7.11 vs. 9.3 or 46.8 vs. 49 respectively for *U*=0.02, 0.2 or 1, Fig. 3a, c & e).

**Fig. 4:**
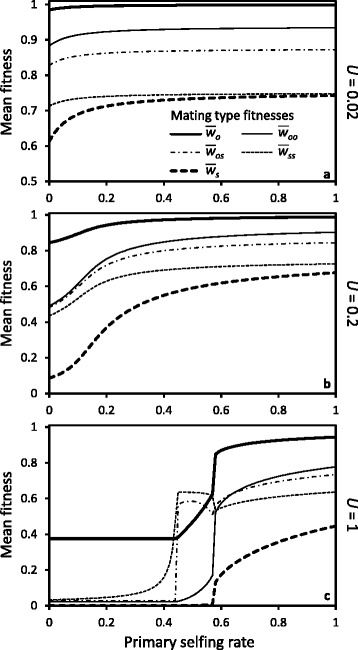
Mean fitness of offspring produced by the types of mating as a function of population selfing rate for different genomic mutation rates to lethals *U* (panels **a**, **b** & **c**), with a sib-mating rate of *b*=0.05 and a background inbreeding depression of *d*=0.25. The types of mating are outcrossing (*o*), selfing (*s*), and sib-mating between two outcrossed individuals (*oo*), one outcrossed and one selfed individual (*os*), or two selfed individuals (*ss*)

For the same rate of inbreeding in a population (as in the foregoing example), sib-mating is more efficient than selfing at purging nearly recessive lethal mutations because the probability of producing homozygous lethals in sib-mating is smaller than under selfing, which reduces selective interference among loci [28]. This is reflected in the mean fitnesses of the different types of progeny. Figure 4 shows that the progeny of all sib-mating types generally have mean fitnesses intermediate between the mean fitness of selfed and outcrossed individuals. The only exceptions are for the types of mating *os* and *ss* under a high genomic mutation rate to lethals (*U*=1, Fig. 4c) at selfing rates near the purging threshold (see below for an explanation).

Mean fitness at equilibrium also varies among types of sib-mating. In general the more inbred offspring have lower mean fitness, due mostly to the constant background component of inbreeding depression. However, with a high genomic mutation rate to lethals the difference among different kinds of sib-mating is reduced, and sometimes reversed (see Fig. 4c, where inbred individuals with two selfed parents have the highest mean fitness at selfing rates below the purging threshold). Without background inbreeding depression, this becomes the rule: the most inbred sib-mating types always have the highest mean fitness (cf. Figure 6 in Appendix). Because selfed parents have purged a fraction of their deleterious mutations, the mean fitness of individuals produced by sib-mating is then higher when their parents are selfed vs. outcrossed. The difference is particularly large under a high genomic mutation rate to lethals (*U*=1, Fig. 4c) at selfing rates near the purging threshold ($\bar {s}=0.38$), where biparentally inbred individuals with at least one selfed parent have higher mean fitness than outcrossed individuals, despite being inbred. Near the purging threshold, an appreciable fraction of selfed zygotes survive (i.e. the secondary selfing rate moves away from zero), and purging occurs in a subset of the population. In this situation, the difference in the number of lethals between outcrossed individuals and surviving selfed (purged) individuals is such that the overall heterozygous effects of lethals in outcrosses between unrelated individuals exceeds the (homozygous) lethal effects in *os* and *ss* sib-matings.

With respect to purging recessive lethal mutations, the results of our model of inbreeding depression in mixed-mating populations practising a combination of outcrossing to unrelated individuals, selfing, and sib-mating are consistent with those of [42], although this earlier study was based on a simpler, less realistic model for inbreeding depression. In both models, BI favours the purging of recessive deleterious mutations in predominantly outcrossing populations, and may therefore facilitate the evolution of selfing. In contrast to Uyenoyama’s model, we incorporated zygotic disequilibrium and selective interference among loci, which are magnified under high inbreeding depression. We show that with high genomic mutation rate to recessive lethals, sib-mating lowers the threshold for purging recessive lethal mutations, thereby potentially favouring evolution of high selfing rates.

Could the lower purging threshold caused by sib-mating be observed in experimental data? Winn et al. [45] observed a purging threshold for total inbreeding depression, with substantial inbreeding depression maintained for selfing rates up to 0.8, but the among-species variation in the rate of BI (Fig. 1 and Duminil et al. 2009) may be too small to allow empirical tests of whether BI affects the purging threshold for recessive lethals.

### Evolutionarily stable selfing rates in a population practicing sib-mating

Following Lande and Schemske [27], who showed that complete outcrossing and complete selfing were the only two evolutionarily stable equilibria maintained by the two main genetic forces driving the evolution of selfing (Fisher’s automatic advantage of selfing and inbreeding depression), numerous models were proposed to explain the maintenance of stable mixed mating (reviewed in [16]). Uyenoyama [42] suggested that BI could be one mechanism maintaining stable intermediate selfing rates under moderate inbreeding depression by generating frequency-dependent selection. At higher selfing rates, the automatic advantage of selfing decreases and BI further decreases the automatic advantage of selfing via greater transmission of the genome in the BI portion of the outcrossed matings. However, Uyenoyama [42] assumed no inbreeding depression associated with BI but constant inbreeding depression associated with selfing, to observe stable mixed mating.

We can use the analytical approximation of Lande and Schemske [27] to predict the evolutionarily stable selfing rates expected in our more realistic model that includes inbreeding depression for progeny of sib-mating and that allows inbreeding depression to evolve as a function of the mating system. The approximation states that there is selection for increased selfing if the inbreeding depression is less than 0.5 (the automatic advantage of selfing), and vice versa. Here, the approximation predicts that the only evolutionarily stable selfing rates are complete outcrossing (*δ*>0.5, Fig. 3d, f) or complete selfing (*δ*<0.5, Fig. 3b, d). Porcher and Lande [36] showed that this approximation predicts evolutionarily stable selfing rates accurately with a low to moderate genomic mutation rate to lethals (*U*=0.02−0.2). A full analysis of evolution of selfing is required under high mutation rate to lethals (*U*=1), when the approximation does not apply. However, in general, allowing purging of inbreeding depression generates a strong positive feedback on the evolution of selfing rates, which can outweigh other mechanisms that promote stable intermediate selfing rates in the absence of purging (e.g. [35] for an example with fluctuating inbreeding depression).

### Scope and limitations of the model

To keep the model tractable, we have assumed that the component of inbreeding depression due to nearly additive, mildly deleterious mutations could not be purged. Combining two components of inbreeding depression represents a significant improvement over most theoretical studies about mating system evolution, which generally assume constant total inbreeding depression, inbreeding depression caused by a single locus, or at best model a single component. Assuming a constant (non-evolving) background inbreeding depression is a reasonable first approximation. The contributions of mutations to inbreeding depression and their sensitivity to purging upon selfing depend primarily on their deleterious effect and dominance coefficient [4]. Furthermore, in predominantly outcrossing species, the total inbreeding depression resulting from the classical bimodal distribution of inbred fitness is composed of comparable amounts due to nearly recessive lethal and semi-lethal mutations versus more nearly additive mildly deleterious mutations [8, 20]. For these reasons, nearly-recessive lethals are expected to be most readily purged by selection with inbreeding, and to play the most important role in the coevolution of inbreeding depression and selfing rate. Data from natural populations show that early-acting recessive lethals contribute a substantial fraction of inbreeding depression in outcrossing species but are mostly purged in highly selfing species [20]. In contrast, the component of inbreeding depression due to late-acting more nearly additive mildly deleterious mutations remains nearly constant across population selfing rates [20].

Estimates of the mean dominance coefficient of mildly deleterious mutations in natural populations are generally in the range of 0.2<*h*<0.5 (e.g. [17]) and theoretical analyses show that for such mutations the contribution to inbreeding depression depends only weakly on the selfing rate [4, 36]. However, a small fraction of mildly deleterious mutations may have smaller dominance coefficients and thereby be liable to purging. In addition, we have shown that slow inbreeding, as produced here by sib-mating, is more efficient than selfing for purging nearly recessive lethals. This may also be true for mildly deleterious mutations [12]. A full model of inbreeding depression, including all types of unconditionally deleterious mutations (with a range of selection and dominance coefficients), as well as inbreeding depression due to stabilizing selection on quantitative characters [29], is therefore needed for a comprehensive understanding of the joint dynamics of inbreeding depression and mating systems. The net effect of purging mildly deleterious alleles should nevertheless remain small compared to the purging of nearly-recessive, highly deleterious mutations. Incorporating the purging of mildly deleterious mutations therefore should not alter the main conclusion of our model, which is that sib-mating contributes to purging inbreeding depression, thereby reinforcing the positive feedback on the evolution of selfing rate. In other words, BI, as modelled here by sib-mating, cannot produce a stable mixed-mating system.

Our model also assumes infinite population size and hence cannot address the effect of sib-mating caused by small population size alone. In small populations, demography and drift may interact with mutation, selection and the mating system to alter the dynamics of inbreeding depression and the purging process. For example, fixation of mildly deleterious mutations decreases inbreeding depression [1]. Yet our model should be valid for other mechanisms causing BI in large populations, such as geographic structuring by limited dispersal of seed and pollen (isolation by distance), which is likely to be common in plants [43]. The main result of our model, that sib-mating promotes increased selfing via purging of inbreeding depression, requires that selfing efficiently purges recessive lethal mutations in finite populations. The few theoretical studies examining the joint effects of selfing rates and population size on the dynamics of inbreeding depression all show that for populations of 100 or more individuals, purging by self-fertilization occurs, particularly with highly deleterious mutations with a small dominance coefficient, such as recessive lethals (e.g. [14] for an analytical treatment with a single locus or [4] for individual-based simulations with a Kondrashov-like model). Thus the predictions from infinite population models are consistent with those from simulations of finite populations (compare e.g. [34] vs. [13] for the evolution of self-incompatibility).

## Conclusions

Using a realistic model of inbreeding depression incorporating the bimodal distribution of deleterious mutations and the joint evolution of mating system and inbreeding depression, we have shown that sib-mating promotes the purging of inbreeding depression, but is unlikely to maintain stable intermediate selfing rates. These results, combined with previous work, suggest that genetic mechanisms alone are therefore unlikely to provide general explanations for the maintenance of mixed mating. Factors involved in pollination ecology, including pollen discounting, and pollinator behaviour, interacting with genetic mechanisms of evolving inbreeding depression, provide a more general explanation of the widespread maintenance of intermediate selfing rates in plant populations (e.g. [7, 33, 36]).

## Methods

### Model assumptions and notation

The assumptions are the same as for the simplified [25] model of mixed selfing and random mating for partially recessive lethals developed in [28], except that we want to allow other forms of inbreeding in addition to selfing. The different types of mating are referred to using subscripts without brackets: *o*, *s*, *oo*, *os*, and *ss* describe respectively individuals produced by outcrossing to an unrelated individual, by self-fertilization, and by sib-mating involving two outcrossed parents, one outcrossed and one selfed parent, or two selfed parents (Fig. 1). In the simplified Kondrashov model, inbreeding depression is due to nearly recessive lethal mutations occurring at an infinite number of unlinked loci in an infinite population. The genomic mutation rate to lethals is *U* and their dominance coefficient *h*. Lethal mutations are never homozygous in adults, segregate independently and have identical effects on fitness, such that a diploid population with discrete non-overlapping generations is fully described by the distribution of number of heterozygous lethals per diploid genome in adults. The frequency of adults in generation {*t*} carrying *x* heterozygous lethal mutations is *p*_{*t*}_(*x*). The life cycle begins with reproduction and is followed by mutation, then selection.

The distribution of heterozygous lethals causes inbreeding depression upon selfing, which is defined as the decrease in mean fitness of selfed offspring $\bar {w}_{s}$ relative to those produced by outcrossing between unrelated parents $\bar {w}_{o}$ so that $\delta = 1-\bar {w}_{s}/\bar {w}_{o}$. We included a constant (non-evolving) component of inbreeding depression due to mildly deleterious nearly additive mutations via a background inbreeding depression *d*=0.25 for selfing, as in [33]. In the present model the background inbreeding depression reduces the mean fitness of the different types of mating in proportion to their (neutral) inbreeding coefficients (Eq. 17), *F*_*o*_=0, $F_{s}=\frac {1}{2}$, $F_{oo}=\frac {1}{8}$, $F_{os}=\frac {1}{4}$ and $F_{ss}=\frac {1}{2}$.

For simplicity, a low frequency of sib-mating below 0.1 is assumed, embedded in a background pedigree of mixed mating (selfing and outcrossing), which is consistent with empirical observations. Hence we can neglect the probability of sib-mating occurring in two successive generations in any given lineage in the population. Finally, we also assume that sib-mating occurs only between individuals from the same maternal family, because the most likely cause of sib-mating in many plant populations is fine-scale genetic structure, most of which is attributable to limited seed dispersal in comparison to pollen flow [31]. This excludes any contributions from full-sib mating, except when the maternal parent of two sibs is selfed (sib-mating type *ss*).

We first describe the production of juvenile heterozygous lethal genotypes in generation {*t*} after mutation but before selection on heterozygotes produced by each type of mating. We then complete the life cycle by appropriately weighting the different types of matings and performing the operations of selection on heterozygous viability and normalization of genotype frequencies in the adults.

### Random mating

A proportion of zygotes are produced by random mating (outcrossing to an unrelated individual) as in the Kondrashov model for partially recessive lethal mutations [28].

In the parental generation {*t*−1} the probability that a mature plant with *y* heterozygous lethals produces a gamete with *x* (≤*y*) lethals (before mutation) is $\binom {y}{x}(1/2)^{y} $. Thus in the entire parental population the probability of producing a gamete with *x* lethals (before mutation) is

1 $$ g(x) = \sum_{y=x}^{\infty} p_{\{t-1\}}(y) \binom{y}{x}\left(\frac{1}{2}\right)^{y}.  $$

With an infinite number of loci every new mutation is unique and outcrossing to an unrelated individual in an infinite population never produces homozygosity of lethal alleles. The probability that random mating produces a zygote with *x* heterozygous lethals (before mutation) is then

2 $$ p_{o}^{*} (x) =\sum_{y=0}^{x} g(x-y)g(y)  $$

and after mutation (but before selection on heterozygotes) the fraction of randomly outcrossed juvenile offspring with *x* heterozygous lethals is

3 $$ p_{o}^{**}(x) = \sum_{y=0}^{x} p_{o}^{*} (x-y)\frac{e^{-U}U^{y}}{y!}.  $$

### Self-fertilization

A proportion of individuals are produced by self-fertilization, as in [28]. In the parental generation {*t*−1} the probability that a mature plant with *y* heterozygous lethals produces by self-fertilization a seed with *x* (≤*y*) heterozygous lethals (before mutation) is $\binom {y}{x}(1/2)^{x} (1/4)^{y-x} = \binom {y}{x}(1/2)^{2y-x} $. Thus in the entire parental population the probability of producing a juvenile offspring with *x* lethals (before mutation) is

4 $$ p_{s}^{*}(x) = \sum_{y=x}^{\infty} p_{\{t-1\}}(y) \binom{y}{x}\left(\frac{1}{2}\right)^{2y-x}.  $$

After mutation (but before selection on heterozygotes) the fraction of outcrossed juvenile offspring in generation {*t*} with *x* heterozygous lethals is

5 $$ p_{s}^{**}(x) = \sum_{y=0}^{x} p_{s}^{*} (x-y)\frac{e^{-U}U^{y}}{y!}.  $$

### Outcrossing between relatives

#### Transmission of lethals from grandparents to offspring

To derive the number of homozygous lethals produced by sib-mating we extend the Kondrashov model to describe the transmission of mutations from grandparents to offspring. Depending on the type of sib-mating (Fig. 1), we distinguish four types of grandparents (from single to quadruple), differing in the number of genome copies they contribute to the parental and offspring generation. Matings in the grandparental generation {*t*−2} that produce half-sibs in generation {*t*−1} cause no homozygosity until the offspring generation {*t*} and therefore have the same distribution of number of heterozygous lethals in the parental generation {*t*−1} as under mixed outcrossing and selfing (*o* and *s*). The probabilities $g^{{~}^{\prime }}(x)$, $g^{{~}^{\prime \prime }}(x)$, $g^{{~}^{\prime \prime \prime }}(x)$, and $g^{{~}^{\prime \prime \prime \prime }}(x)$, that in the entire population at generation *t*−2, a single, double, triple or quadruple grandparent, respectively, produces a juvenile offspring in generation *t* with *x* heterozygous lethals, are derived in the Appendix. These relative probabilities sum to less than 1, either because parents that die due to heterozygous lethals make no contribution to the offspring generation or because offspring die due to homozygous lethals produced by sib-mating. Thus these formulae account for selection on heterozygous lethals in parents, and selection on homozygous lethals in offspring, but do not include any selection on heterozygous lethals in offspring, which we deal with afterwards.

#### Convolution of grandparental contributions

In a half-sib mating involving two outcrossed individuals (*oo*), three unrelated grandparents may contribute heterozygous lethals to the offspring of each half-sib mating. The three contributions result from independent events involving genetic segregation and recombination. Each grandparent outcrosses to an unrelated individual, with the double grandparent outcrossing to two different unrelated individuals. The total contribution to heterozygous lethals in juvenile offspring from the three grandparents is simply the sum from the three grandparents, so the unnormalized distribution of heterozygous lethals from these three sources is simply the convolution of the distributions from these three sources. This can be expressed in two concatenated convolutions, first convoluting the two single grandparent contributions,

6 $$ g^{\prime*}(z)=\sum_{x=0}^{z}g'(z-x)g'(x)  $$

and then convoluting this with the double grandparent distribution to get the unnormalized distribution of total number of heterozygous lethals contributed by the grandparents,

7 $$ p_{oo}(x)=\sum_{z=0}^{x}g^{\prime\prime}(x-z)g^{\prime*}(z).  $$

In a sib-mating involving one selfed and one outcrossed individuals, only two unrelated grandparents (one single, one triple) may contribute heterozygous lethals to the offspring of each half-sib mating. The distribution of heterozygous lethals in the offspring is therefore a convolution from these two grandparents:

8 $$ p_{os}(x)=\sum_{z=0}^{x}g^{\prime\prime\prime}(x-z)g'(z).  $$

Finally, in a sib-mating involving a quadruple grandparent, the distribution of heterozygous lethals in the offspring is given directly by Eq. (21)

9 $$ p_{ss}(x)=g^{\prime\prime\prime\prime}(x)  $$

#### New mutations in parents

Now we must account for the extra generation of mutation and selection on the new mutations produced during reproduction by grandparents and passed to juveniles of the parental generation. New mutations expressed in juvenile parents in generation {*t*−1} are inherited and selected only in heterozygotes and are therefore unaffected by inbreeding (from all grandparental sources). On average, *U* new mutations arise in each of two parents, a fraction $\frac {1-h}{2}$ of which are transmitted to the offspring, accounting for selection on heterozygous lethals and genetic segregation. Hence, the total mutation rate is *U*(1−*h*) and the fitness of each parent is reduced by a factor *e*^−*Uh*^, regardless of their mutation load. For sib-mating type *ij* (where *ij*=*oo,os* or *ss*)

10 $$ p_{ij}^{*}(x)=e^{-2Uh}\sum_{y=0}^{x}p_{ij}(x-y)\frac{e^{-U(1-h)}\left[U(1-h)\right]^{y}}{y!}  $$

#### New mutations in the offspring

For all types of mating, the relative frequency of juvenile offspring produced by half-sib mating containing *x* heterozygous lethal mutations after mutation (but before selection) is

11 $$ p_{ij}^{**}(x)=\sum_{y=0}^{x}p_{ij}^{*}(x-y)\frac{e^{-U}U^{y}}{y!}.  $$

#### Normalization to account for selection in parents

The sum over *x* of $p_{ij}^{**}(x)$ frequencies calculated above is less than 1, due to selection on homozygous and heterozygous lethals in the parental generation, and selection on homozygous lethals in the offpsring generation. A renormalization is therefore necessary to correct for selection in the parental generation, and combine the frequencies of individuals produced by sib-mating (calculated over two generations) with those of outcrossed and selfed individuals (calculated over a single generation). Because crosses do not occur at random, the normalization factor for each type of mating *ij* is not a simple product of population mean fitnesses, but instead the mean fitness of all possible pairs of sib parents within the type of mating

12 $${} \begin{aligned} \bar{w}_{ij}&=e^{-2Uh}\sum_{x=0}^{\infty} p_{\{t-2\}}(x)\\ &\quad\sum_{y_{1}}\sum_{y_{2}} (1-h)^{y_{1}+y_{2}}q_{i\{t-1\}}(x,y_{1})q_{j\{t-1\}}(x,y_{2}) \end{aligned}  $$

where *q*_*i*{*t*−1}_(*x,y*) is the probability that a grandparent carrying *x* heterozygous lethals produces a parent with *y* heterozygous lethals via outcrossing (*i*=*o*) or selfing (*i*=*s*). The factor *e*^−2*Uh*^ allows mutation decreasing the fitness of the parental generation. It can be shown that

13a $$\begin{array}{@{}rcl@{}} q_{o\{t-1\}}(x,y)&=&\sum_{k=0}^{min(x,y)} \binom{x}{k}\left(\frac{1}{2}\right)^{x} g'(y-k) \end{array} $$

13b $$\begin{array}{@{}rcl@{}} q_{s\{t-1\}}(x,y)&=& \binom{x}{y}\left(\frac{1}{2}\right)^{y}\left(\frac{1}{4}\right)^{x-y} \end{array} $$

Using Eqs. (13) in Eq. (12), we obtain the following normalization factors

14a $$\begin{array}{@{}rcl@{}} \bar{w}_{oo}&=&e^{-Uh}\bar{w}_{o} \sum_{x=0}^{\infty} p_{\{t-2\}}(x) \left(1-\frac{h}{2}\right)^{2x} \end{array} $$

14b $$\begin{array}{@{}rcl@{}} \bar{w}_{os}&=&e^{-3Uh/2}\sqrt{\bar{w}_{o}} \sum_{x=0}^{\infty} p_{\{t-2\}}(x) \left[\left(1-\frac{h}{2}\right)\left(\frac{3-2h}{4}\right)\right]^{x}\\ \end{array} $$

14c $$\begin{array}{@{}rcl@{}} \bar{w}_{ss}&=&e^{-2Uh} \sum_{x=0}^{\infty} p_{\{t-2\}}(x) \left(\frac{3-2h}{4}\right)^{2x} \end{array} $$

and we have

15 $$ p_{ij}^{***}(x)=\frac{p_{ij}^{**}(x)}{\bar{w}_{ij}}  $$

Note that the constant background inbreeding depression, *d*, does not appear in the normalization factors, although it does affect the mean fitness of types of mating involving at least one selfed parent, reducing it by a multiplicative factor (1−*d*) for each selfed parent. This same multiplicative factor also alters the probability of transmission of lethal alleles from grandparents to offspring detailed in the Appendix, such that they cancel out in the normalization step and are not shown.

### Selection and combination of types of mating in generation *t*

#### Relative frequencies of the different types of mating

Assuming that the frequencies of different types of sib-matings are not genetically determined in the population, then the relative frequencies of types *oo*, *os* and *ss* sib-matings respectively are proportional to the secondary rates (after selection) of random mating and selfing (respectively $1-\bar {s}^{*}$ and $\bar {s}^{*}$). Assuming that the primary selfing rate (at fertilization), $\bar {s}$, is the principal evolutionary variable, so that if $\bar {s}$ increases the other rates must decrease proportionally, all other rates are proportional to $1-\bar {s}$. In addition, we assume a metapopulation-like dispersal model, with short distance seed dispersal generating family “islands”, and both short (within-family, rate *b*) and long-distance (between families, rate 1−*b*) pollen dispersal. Hence, half-sib mating rate is controlled by the constant parameter *b*, i.e. the probability that a plant is pollinated by local pollen produced by a sib. Each family consists of a fraction $\bar {s}^{*}$ of selfed adult plants and a fraction $1-\bar {s}^{*}$ of outcrossed adult plants. The relative probabilities of each type of mating are thus

- Selfing rate, $f_{s} = \bar {s}_{\{t\}}$
- Outcrossing to unrelated individual, $f_{o} = (1-\bar {s}_{\{t\}})(1-b)$
- Half-sib type *oo*, $f_{oo} = \left (1-\bar {s}_{\{t\}}\right)b\left (1-\bar {s}^{*}_{\{t-1\}}\right)^{2}$
- Half-sib type *os*, $ f_{os} = 2\left (1-\bar {s}_{\{t\}}\right)b\bar {s}^{*}_{\{t-1\}}\left (1-\bar {s}^{*}_{\{t-1\}}\right)$
- Full-sib type *ss*, $ f_{ss} = \left (1-\bar {s}_{\{t\}}\right)b\bar {s}^{*2}_{\{t-1\}}$

where $\bar {s}^{*}_{\{t-1\}}=\bar {s}\bar {w}_{s\{t-1\}}/\bar {w}_{\{t-1\}}$ is the secondary selfing rate in the parental generation (see below for definitions of $\bar {w}_{s\{t-1\}}$ and $\bar {w}_{\{t-1\}}$). This gives *f*_*s*_+*f*_*oo*_+*f*_*os*_+*f*_*ss*_+*f*_*o*_=1. To conform to the assumptions, we must restrict *b*<<1.

#### Selection and final step of normalization

The normalization of the total frequency of recessive lethals in mature offspring, incorporating selection on heterozygous lethals and the constant, late-acting component of inbreeding depression due to mildly deleterious alleles, *d*, is

16 $$  p_{\{t\}}(x)=\frac{(1-h)^{x} }{\bar{w}_{\{t\}}}\sum_{r} (1-2dF_{r})f_{r} p_{r}^{***}(x)  $$

where the sum includes all the mating types *r*, with *F*_*r*_ being the neutral inbreeding coefficient of each mating type (given at beginning of Methods) and the mean fitness $\bar {w}_{\{t\}}=\sum _{r} f_{r}\bar {w}_{r\{t\}}$ is the weighted average of the mean fitnesses of progenies produced by the different matings,

17 $$  \bar{w}_{r\{t\}}=(1-2dF_{r}) \sum_{x=0}^{\infty} (1-h)^{x} p_{r}^{***}(x)  $$

### Numerical analysis

The variables are the same as in the Kondrashov model for selfing, but to perform the iterations we must keep track of the last two generations of *p*(*x*), instead of just the single previous generation. Numerical iterations start with one generation of mixed selfing and outcrossing, thereafter allowing also a low rate of sib-mating.

Parameter values are as follows. The genomic mutation rate to lethals is varied between *U*=0.02 and *U*=1, to encompass the limited number of experimental estimates. Data from Drosophila [2] and annual plants [23] indicate genomic mutation rates to lethals on the order of *U*=0.01−0.03. Few estimates exist for large perennial or partially asexual plants, in which the mutation rate to lethals may be much higher due to accumulation of somatic mutations. For example, Lande et al. [28] extrapolated mutation rates to (lethal) embryonic chlorophyll deficiency to the whole genome to obtain a genomic mutation rate of *U*=0.2 in a long-lived mangrove tree. Similarly, high genomic mutation rates to lethal (*U*=0.2 and above) are needed to explain inbreeding depression close to 1 in large gymnosperm trees [18, 37]. Although *U*=1 may exceed the maximum actual genomic mutation rate to lethals, this value illustrates the impact of selective interference in purging partially recessive deleterious mutations caused by high inbreeding depression; selective interference has been demonstrated to occur even with mildly deleterious mutations [24].

The dominance coefficient of lethals is always set to *h*=0.02 [40]. The rate of sib-mating is varied between *b*=0 and *b*=0.1, conforming to assumptions and experimental estimates (Fig. 2). Finally, the constant background component of inbreeding depression is set to *d*=0.25, which is close to the average level of late-acting inbreeding depression that is not purged in highly selfing populations [20].

### Inference of evolutionarily stable selfing rates

To examine the effect of sib-mating on the evolution of selfing rates, we used an approximation first proposed by Lande and Schemske [27] and later generalized by Porcher and Lande [36] to include ecological mechanisms. This approximation assumes that the mating system evolves by small infrequent mutational steps and allows the equilibrium inbreeding depression to evolve as a function of the mating system. It can be used to find the joint equilibria of selfing rate and inbreeding depression by examining the indirect selection gradient on small changes in the selfing rate. Equilibrium selfing rates occur when the selection gradient is 0, when total inbreeding depression exactly counterbalances all other constraints on the evolution of selfing. With selfing and outcrossing alone, the other constraint is the automatic genetic advantage of inbreeding: 50 *%* for selfing [11], so that an unstable evolutionary equilibrium of the selfing rate occurs at *δ*=0.5. The inclusion of sib-mating diminishes the automatic advantage of selfing by increasing gene transmission in other types of mating and also modifies the total inbreeding depression. Because we assume a small rate of sib-mating it does not qualitatively change the stability of the unstable evolutionary equilibrium for the selfing rate, and it produces only a small change in the position of the unstable equilibrium (see Appendix).

**Fig. 5:**
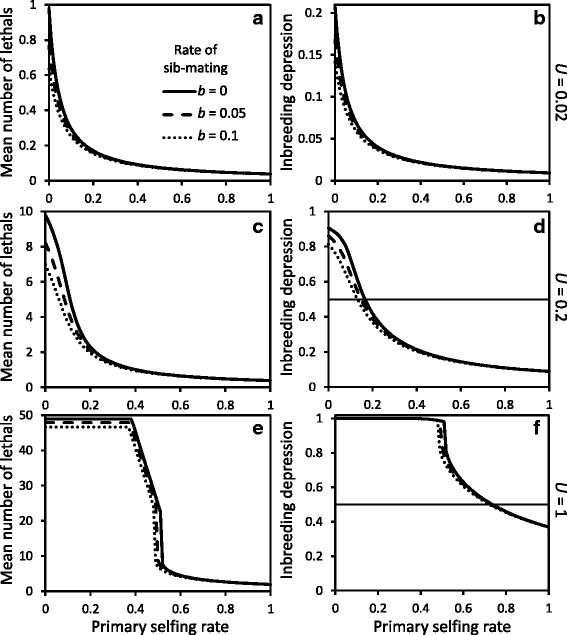
Mean number of heterozygous lethals at equilibrium (**a**, **c** & **e**) and average inbreeding depression (**b**, **d** & **f**) as a function of population selfing rate, for different rates of sib-mating *b* and genomic mutation rates to lethals *U*. There is no background inbreeding depression (*d*=0). On panels **d** and **f**, the thin horizontal line at 0.5 represents the automatic advantage of selfing, i.e. the threshold value for inbreeding below which evolution of increased selfing is favored

## Appendix

### Transmission of lethal alleles from grandparents to offspring

#### Single grandparent

For each heterozygous lethal allele in a single grandparent the relative probability that the juvenile offspring of half-sib matings inherits a heterozygous lethal from them is $\frac {1-h}{4}$. The relative probability that the juvenile offspring does not inherit a heterozygous lethal from one of its single grandparents is $\frac {1}{2} +\frac {1-h}{4} = \frac {3-h}{4}$. Hence, if the single grandparent carries *y* heterozygous lethals, the relative probability of the juvenile offspring of half-sib mating inheriting *x* of these in heterozygous form is $\binom {y}{x}\left (\frac {1-h}{4}\right)^{x} \left (\frac {3-h}{4}\right)^{y-x}$. Averaging over all possible single grandparents the relative probability that a single grandparent contributes *x* heterozygous lethals to the juvenile offspring of half-sib mating is

18 $$  g'(x)=\sum_{y=x}^{\infty} p_{\{t-2\}}(y)\binom{y}{x}\left(\frac{1-h}{4}\right)^{x} \left(\frac{3-h}{4}\right)^{y-x}  $$

where the subscript {*t*−2} refers to the grandparental generation.

#### Double grandparent

For each lethal heterozygote in a double grandparent, the juvenile offspring of half-sib mating is heterozygous for the lethal allele with relative probability $2\left (\frac {1-h}{4}\right)\left (\frac {3-h}{4}\right)$, and is homozygous for non-lethal allele(s) with relative probability $\left (\frac {3-h}{4}\right)^{2}$. Thus, given a double grandparent with *y* heterozygous lethals, the relative probability that the juvenile offspring of a half-sib mating inherits *x* (≤*y*) heterozygous lethals from the double grandparent is $\binom {y}{x}\left (\frac {1-h}{2}\right)^{x} \left (\frac {3-h}{4}\right)^{2y-x}$. Averaging over all common grandparental contributions to half-sib matings, the unnormalized distribution of number of heterozygous lethals inherited from double grandparents is

19 $$ g^{\prime\prime}(x)=\sum_{y=x}^{\infty}p_{\{t-2\}}(y)\binom{y}{x}\left(\frac{1-h}{2}\right)^{x} \left(\frac{3-h}{4}\right)^{2y-x}.  $$

#### Triple grandparent

Given a triple grandparent in generation {*t*−2} with a heterozygous lethal allele at a particular locus, when producing an outcrossed (half-sib) parent in generation {*t*−1}, the parent is heterozygous for this allele with probability $\frac {1-h}{2}$, and does not contain this allele with probability $\frac {1}{2}$. Similarly, the relative probability that a selfed (half-sib) parent in generation {*t*−1} is heterozygous for this allele is $2\left (\frac {1-h}{2}\right)\frac {1}{2} =\frac {1-h}{2}$, and the relative probability that the selfed parent does not contain this allele is $\frac {1}{4}$.

Pairing one selfed and one outcrossed half-sib parents, the relative probability that in generation {*t*} the juvenile offspring of type *os* half-sib mating carry a heterozygous lethal at this locus is therefore $ P=\frac {2-h}{4}\frac {1-h}{4}+\frac {3-h}{4}\frac {1-h}{4} = \frac {(1-h)(5-2h)}{16}. $ Similarly, the relative probability that the juvenile offspring in generation {*t*} lack the lethal allele is $Q= \frac {(2-h)(3-h)}{16}.$ The relative probability that a triple grandparent containing *y* heterozygous lethals produces juvenile offspring with *x* (≤*y*) heterozygous lethals is $\binom {y}{x}P^{x} Q^{y-x}$. Therefore in the entire population in generation {*t*−2} the probability that a triple grandparent produces a juvenile offspring in generation {*t*} with *x* heterozygous lethals (before mutation) is

20 $$ g^{\prime\prime\prime}(x)=\sum_{y=x}^{\infty}p_{\{t-2\}}(y)\binom{y}{x}P^{x} Q^{y-x}.  $$

**Fig. 6:**
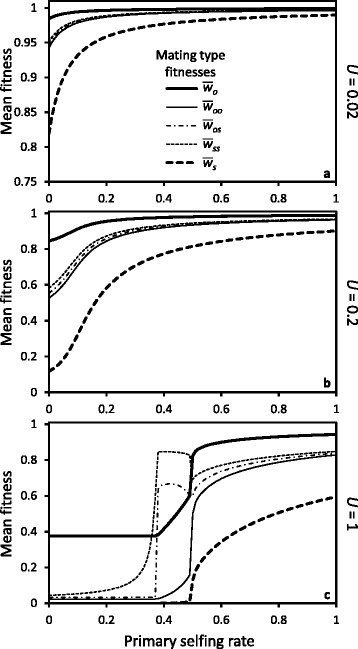
Mean fitness of offspring produced by the types of mating as a function of population selfing rate for different genomic mutation rates to lethals *U* (panels **a**, **b** & **c**), with a sib-mating rate of *b*=0.05 and no background inbreeding depression (*d*=0). The types of mating are outcrossing (*o*), selfing (*s*), and sib-mating between two outcrossed individuals (*oo*), one outcrossed and one selfed individual (*os*), or two selfed individuals (*ss*)

#### Quadruple grandparent

Given one quadruple grandparent in generation {*t*−2} with a heterozygous lethal allele at a particular locus, the relative probability that a selfed (half-sib) parent in generation {*t*−1} is heterozygous for this allele is $\frac {1-h}{2}$, and the relative probability that the selfed parent does not contain this allele is $\frac {1}{4}$.

Thus the relative probability that in generation {*t*} the juvenile offspring of type *ss* full-sib mating carry a heterozygous lethal at this locus is $ P'=2\frac {2-h}{4}\frac {1-h}{4}=\frac {(1-h)(2-h)}{8} $ and the relative probability that the juvenile offspring in generation {*t*} lack the lethal allele is $ Q'= \frac {(2-h)^{2}}{16}. $ The relative probability that a quadruple grandparent containing *y* heterozygous lethals produces juvenile offspring with *x* (≤*y*) heterozygous lethals is $\binom {y}{x}P'^{x} Q'^{y-x}$. Therefore in the entire population in generation {*t*−2} the probability that a quadruple grandparent produces a juvenile offspring in generation {*t*} with *x* heterozygous lethals (before mutation) is

21 $$  g^{\prime\prime\prime\prime}(x)=\sum_{y=x}^{\infty}p_{\{t-2\}}(y)\binom{y}{x}P'^{x} Q'^{y-x}.  $$

**Fig. 7:**
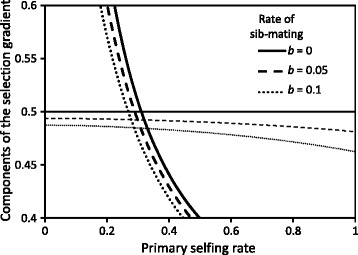
Inference of approximate evolutionary equilibrium selfing rates with sib-mating. The two components of the selection gradient on a modifier with a small effect on the selfing rate (Eq. 26) are plotted against the primary selfing rate for different rates of sib-mating *b*: automatic advantage of selfing (thin lines) and total inbreeding depression (thick lines) in the population, including selfing and sib-mating. The vertical scale is expanded so that the small differences in the automatic advantage and in the total inbreeding depression across rates of sib-mating are visible. The genomic mutation rate to lethals is *U*=0.2 and the background inbreeding depression upon selfing is *d*=0.25

### Approximation for evolution of selfing under low rates of sib-mating

The approximation assumes an infinite population with primary selfing rate $\bar {s}$ in which an initially rare modifier with selfing rate *s* appears [27]. Assuming that all types of mating produce the same amount of pollen, and that all ovules are fertilized, the expected fitness of individuals with the selfing rate *s*, without sib-mating, is:

22 $$ w=s\bar{w}_{s}+\frac{1}{2}(1-s)\bar{w}_{o}+\frac{1}{2}(1-\bar{s})\bar{w}_{o}  $$

Selfed offspring are weighted twice as much as outcrossed offspring to incorporate the automatic genetic advantage of selfing. The intensity of selection on a modifier with a small effect on the selfing rate is approximately proportional to the selection gradient:

23 $$ \frac{1}{\bar{w}_{o}}\frac{\partial w}{\partial s}=\frac{\bar{w}_{s}}{\bar{w}_{o}}-\frac{1}{2}=\frac{1}{2}-\delta   $$

Evolutionary equilibrium selfing rates occur when the selection gradient is zero, which, without sib-mating, amounts to comparing inbreeding depression with the automatic advantage of selfing. Because the equilibrium inbreeding depression *δ* is a decreasing function of the population selfing rate, provided that *δ*>1/2 in an outcrossing population and *δ*<1/2 in a completely selfing population, an unstable evolutionary equilibrium selfing rate occurs at an intermediate value, $0<\bar {s}<1$ (see Fig. 3).

With sib-mating, the weighting of outcrossed vs. selfed offspring changes, because individuals reproducing via BI transmit more than one copy of their genome. The fraction of the genome of an individual mating with a relative that is found in its offspring is 5/8, 11/16 and 7/8, respectively for sib-mating types *oo*, *os* and *ss*, respectively (vs. 1/2 for outcrossing between unrelated individuals and 1 for selfing). Therefore,the expected fitness of individuals with the selfing rate *s* becomes:

24 $$ \begin{aligned} w&=s\bar{w}_{s}+(1-s)\left[(1-b)\frac{1}{2}\bar{w}_{o}+b\left((1-\bar{s}^{*})^{2}\frac{5}{8}\bar{w}_{oo}\right.\right.\\&\quad+ \left.\left.2\bar{s}^{*}(1-\bar{s}^{*}) \frac{11}{16}\bar{w}_{os}+\bar{s}^{*2}\frac{7}{8}\bar{w}_{ss}\right)\right]+P(\bar{s},\bar{s}^{*}) \end{aligned}  $$

where $\bar {s}^{*}$ is the secondary selfing rate after selection in the population and $P(\bar {s},\bar {s}^{*})$ is the siring success of individuals with selfing rate *s* which depends solely on the population selfing rate ($\bar {s}$ and $\bar {s}^{*}$) and disappears when fitness is differentiated with respect to *s*. The selection gradient is:

25 $$ \begin{aligned} \frac{1}{\bar{w}_{o}}\frac{\partial w}{\partial s}&=\frac{\bar{w}_{s}}{\bar{w}_{o}}-\frac{1-b}{2}-b\left((1-\bar{s}^{*})^{2}\frac{5}{8}\frac{\bar{w}_{oo}}{\bar{w}_{o}}\right.\\&\quad+\left.2\bar{s}^{*}(1-\bar{s}^{*}) \frac{11}{16}\frac{\bar{w}_{os}}{\bar{w}_{o}}+\bar{s}^{*2}\frac{7}{8}\frac{\bar{w}_{ss}}{\bar{w}_{o}}\right) \end{aligned}  $$

This can be rewritten :

26 $${} \begin{aligned} \frac{1}{\bar{w}_{o}}\frac{\partial w}{\partial s}&=\frac{1}{2}+b\left[\frac{1}{2}-\left((1-\bar{s}^{*})^{2}\frac{5}{8}+2\bar{s}^{*}(1-\bar{s}^{*})\frac{11}{16}+\bar{s}^{*2}\frac{7}{8}\right)\right]\\ &\quad-\left[\!\delta -b\left(\!\!(1-\bar{s}^{*})^{2}\frac{5}{8}\delta_{oo}+2\bar{s}^{*}(1-\bar{s}^{*})\frac{11}{16}\delta_{os}+\bar{s}^{*2}\frac{7}{8}\delta_{ss} \!\!\right)\!\right] \end{aligned}  $$

where *δ*_*oo*_, *δ*_*os*_ and *δ*_*ss*_ are the inbreeding depressions of sib-mating types *oo*, *os* and *ss*, defined as 1 minus the ratio of their mean fitness over the mean fitness of offspring produced by outcrossing between unrelated parents. The two parts of the right side can be compared with those of Eq. (23). The first part (first two terms) corresponds to the automatic transmission advantage of selfing, now decreased by sib-mating; the increased transmission of genes by sib-mating favours outcrossing genes, which are more concentrated in sib-matings than in selfed matings. The second part (third term) is the total inbreeding depression in the population, modified by sib-mating, in a complicated way because sib-mating also contributes to the purging of lethals. With rates of sib-mating below 10 *%*, all effects remain small, on the order of a few percent (compare Figure 7 in Appendix with Fig. 3d). Most importantly, incorporating sib-mating only displaces slightly the location of the unstable equilibrium selfing rate, when it exists, but never creates intermediate stable selfing rates, because total inbreeding depression remains a decreasing function of the primary selfing rate, always decreasing faster than the automatic advantage.
